# Retrospective review of outcomes in patients with DNA-damage repair related pancreatic cancer

**DOI:** 10.1186/s13053-020-00148-9

**Published:** 2020-08-10

**Authors:** Sarah K. Macklin-Mantia, Stephanie L. Hines, Pashtoon M. Kasi

**Affiliations:** 1 Department of Clinical Genomics, Mayo Clinic, Jacksonville, FL 32224 USA; 2 Department of Diagnostic and Consultative Medicine, Mayo Clinic, Jacksonville, FL 32224 USA; 3 Holden Comprehensive Cancer Center, University of Iowa, 200 Hawkins Drive, Iowa City, IA 52242 USA

**Keywords:** Hereditary cancer, Pancreatic cancer, Platinum chemotherapy, PARP inhibitors, BRCA1/2, Genetic testing

## Abstract

**Background:**

Patients with DNA-damage response genes (DDR)-related pancreas cancer (*BRCA1/2* or other DNA-damage related genes) may have improved outcomes secondary to increased sensitivity to DNA-damaging drugs (platinum chemotherapy/ poly ADP ribose polymerase (PARP)-inhibitors). However, data is scarce pertaining to outcomes in this subset of patients. Our objective was to retrospectively identify DDR-related pancreas cancer patients and report on clinical outcomes.

**Methods:**

Pancreas cancer patients with a germline pathogenic variant in *BRCA1/2* or other DDR gene were identified retrospectively through review of medical records (medical genetics/oncology) and genetic testing results at our institution. Data regarding clinical outcomes, therapy received, and survival was subsequently extracted.

**Results:**

A total of 11 patients with pancreas cancer were identified to carry a pathogenic DDR-variant: *BRCA1* (3), *ATM* (4), *BRCA2* (2), *PALB2* (1) and *FANCC* (1). Five of these individuals had prior history of other cancers. Clinically these tumors were localized (4), locally advanced (3), and metastatic (4) at diagnosis. Four out of 11 patients were still alive at time of data review. Survival in the 7 patients who had died was 13.7, 140.0, 20.5, 22.3, 23.5, 25.8, and 111.5 months. All patients with advanced disease had exposure to platinum chemotherapy.

**Conclusions:**

Historical survival in patients with advanced and metastatic pancreas cancer is poor. Results of this DDR-subset of patients do show significantly superior outcomes, likely secondary to exposure to platinum drugs. This data, alongside other similar cohorts, would favor the DDR-genes being a predictive marker with improved survival if exposed to these drugs and the new class of drugs, PARP-inhibitors.

## Background

For years, germline testing for hereditary cancer syndromes was completed largely to provide guidance for future surveillance and provided little to no clinical utility for those already affected with pancreatic cancer. Developments in our understanding of pancreatic cancer pathology opened additional applications for genetic results. Individualized approaches for pancreatic adenocarcinoma receive keen attention as survival rates are among the poorest, with 5-year survival around 8% [1]. Germline and somatic results can influence management recommendations and possibly general prognosis as well.

Treatment alterations can be considered if a cancer shows mismatch repair (MMR) or DNA damage repair (DDR) deficiency [2–5]. Individuals with a DDR-related cancer can include those with a pathogenic, germline or somatic *BRCA1/2* variant and other genes within the homologous recombination and Fanconi anemia pathways. This population appears to have better outcomes compared to the general pancreatic cancer population. Median all stage overall pancreatic cancer survival has been reported as 14 months for those with a pathogenic *BRCA1/2* variant [6]. For reference, those with metastatic pancreatic cancer generally have an estimated median survival below 6 months [7, 8]. Following clear margin removal of a pancreatic tumor, median survival time increases to around 23 months [8, 9]. Prognosis between those with a *BRCA1/2-*related pancreatic cancer and those with an apparently sporadic cancer may be more similar if both tumors are resectable [10].

DDR-related pancreatic tumors also appear to have a better response to platinum- based regimens and/or PARP inhibitors [2, 6, 11]. Stage 3 or 4 pancreatic cancer survival increased from 9 to 22 months for those with a *BRCA1/2* mutation (*P* = 0.039) if a platinum-based chemotherapy was introduced into their care [6]. Another study reported median time of survival of 11 months in the *BRCA1/2*- population (95% CI, 1.5–12) and 23.3 months in *BRCA1/2*+ group (95% CI, 3.8–30.2) with cisplatin, gemcitabine and veliparib [2]. Others found median survival was 46.6 months for those with a pathogenic *BRCA1/2* or *PALB2* variant following platinum exposure compared to 23.3 for those without a variant detected [12].

## Methods

This clinical review was approved by the Mayo Clinic Florida Institutional Review Board (ID:18–006620). The clinical histories of patients with pancreatic adenocarcinoma and a germline pathogenic variant in a hereditary cancer gene were retrospectively reviewed. Patients were identified by the Mayo Clinic Florida Clinical Genomics Department and the Division of Oncology between 2016 and 2018. These patients had not only imaging to confirm their pancreatic cancer diagnoses, but also pathology analysis confirming adenocarcinoma. Patients had been referred to the Clinical Genomics Department due to personal history, family history, and/or a somatic genetic test result suggestive of a hereditary cancer syndrome in accordance with the standard of care for genetic testing at the time. Patients underwent germline genetic testing through various CAP accredited/ CLIA certified commercial genetic testing companies.

## Results

Eleven patients with pancreatic cancer were found to carry a hereditary cancer risk. The average age of pancreatic cancer diagnosis of this population was 60.3 years (SD = 15.9). All patients were Caucasian, aside from Patient 11 who was African American. Patient 5 reported possible Ashkenazi Jewish ancestry. Five patients had prior history of cancer [Table 1]. Patients 3, 4, and 11 had a breast cancer diagnosis prior to age 50. Patients 2–6, 8, 9, and 11 had at least 1 first degree relative with pancreatic, breast, ovarian, or prostate cancer, and 6 of those patients had at least 2 of those diagnoses in first degree relatives. In most cases, it was not possible to determine whether the variants had been maternally or paternally inherited. Pathogenic variants detected were within *BRCA1* [2], *ATM* [3], *BRCA2* [4], *PALB2* [1] and *FANCC* [1]. Three individuals had variants of uncertain significance (VUSs) reported. Patient 11 had a VUS in *PMS2*, and Patient 5 had 1 in *POLE*. Patient 3 had a VUS in *RAD50*, *RAD51C*, and *SDHB*. While some variants had initially been detected through a somatic focused test, all were confirmed to be present in the germline DNA.

**Table 1:** Clinical History of Patients with a DDR- related Pancreatic Cancer

Pt	Sex	Prior cancer	Family history^h^	Dx. age	Clinical stage at dx.	Gene	Variant	Survival (mo.)	Sx.	Chemotherapy	
										Drug	Duration (mo.)
1	M	–	FDR: Colon (P)	30–35	metastatic	*BRCA1*	c.34C > T	25.8	No	FOLFIRINOX	5
			SDR: Breast (P), Prostate (M), Renal (M)							Erlotinib	1
										Gemcitabine, Nab-Paclitaxel	6
2	M	–	FDR: Pancreas (P), Prostate (P), Uterine (M)	55–60	metastatic	*BRCA1*	c.5080G > T	23.5	Yes^a^	FOLFIRINOX	3
										Gemcitabine, Nab-Paclitaxel	2
										Gemcitabine	2
										Gemcitabine, Cisplatin	7
3	F	breast	FDR: Breast (M), Colon (P)	40–45	metastatic	*ATM*	c.2921 + 1G > A	13.7	No	FOLFIRINOX	8
			SDR: Colon (P)								
4	F	bilateral breast	FDR: Breast (M), Colon (P), Ovarian (M)	50–55	locally advanced	*BRCA1*	c.2722G > T	111.5	Yes	Gemcitabine	6
										FOLFIRINOX	6
			SDR: 3 Breast (P)							Gemcitabine, Nab-Paclitaxel	1
										Gemcitabine	8
										FOLXFOX	6
										Irinotecan	3
										Nivolumab	13
										Nivolumab, Gemcitabine, Carboplatin	1
										Nivolumab, Gemcitabine	2
										Nivolumab	1
										PARPi (Rucaparib)	1
5	F	–	FDR: Pancreas (M), Prostate (P)	60–65	metastatic	*ATM*	c.7630-2A > C	20.5	No	FOLFIRINOX	14
			SDR: Breast (M), 2 Pancreas (M), Prostate (P)							Gemcitabine, Cisplatin	4
6	M	–	FDR: Breast (M), Ovarian (M)	60–65	locally advanced	*BRCA2*	c.9435_9436del	34.3^b^	Yes^a^	FOLFIRINOX	2
			SDR: 2 Breast (P)							Gemcitabine, Capecitabine	5
7	M	basal cell carcinoma	SDR: Bladder (M), Breast (P), Colon (P)	70–75	locally advanced	*FANCC*	c.1642C > T	22.3	No	Gemcitabine, Nab-Paclitaxel	4
										FOLFIRINOX	7
8	F	–	FDR: Breast (M), Melanoma (M)	40–45	localized	*PALB2*	c.487_488delGT	84.4^b^	Yes	Gemcitabine	5
			SDR: Breast (M)								
9	F	melanoma, bladder	FDR: Breast (M), Melanoma, 2 Pancreatic, Prostate (P)	80+	localized	*ATM*	c.6975 + 2 T > C	56.6^b^	Yes	Gemcitabine	3
10	M	–	FDR: Cholangiocarcinoma	80+	localized	*ATM*	c.2921 + 1G > A	43.2^b^	Yes	Gemcitabine	2
			SDR: Glioblastoma								
11	F	breast	FDR: Breast (M), Prostate (P)	75–80	localized	*BRCA2*	c.1976_1977insSVA	14.0^c^	Yes	Gemcitabine	3
			SDR: Breast (M), 4 Prostate (M), Prostate (P)								

^a^Resectable following chemotherapy. ^b^These patients have not passed away. ^c^Died due to comorbidities (no recurrence). ^d^FDR: First Degree Relative. ^e^SDR: Second degree relative. ^f^(P): Paternal relative. ^g^(M): Maternal relative. ^h^Unconfirmed cancers/ unconfirmed primaries are not included

**Fig. 1:**
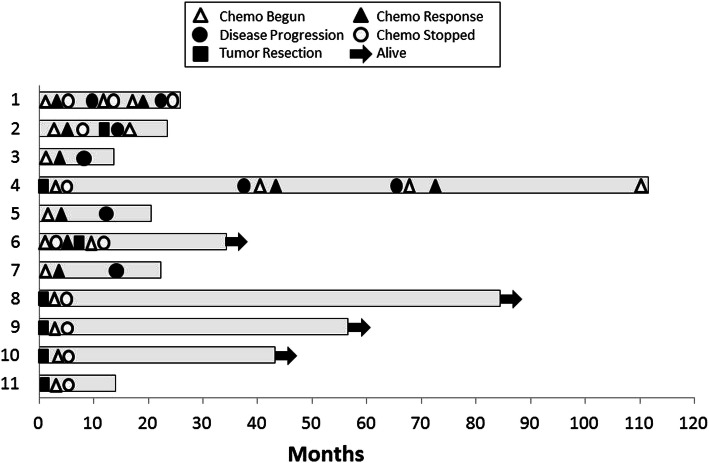
Timeline of survival following diagnosis of pancreatic adenocarcinoma

Four tumors were initially localized, 3 locally advanced, and 4 metastatic. At time of diagnosis, 5 of the tumors discovered were resectable, and 2 were deemed resectable only following chemotherapy. Survival for patients initially diagnosed with either metastatic or advanced disease that had died at the time of clinical review measured 13.7, 20.5, 22.3, 23.5, 25.8, and 111.5 months (Fig. 1). All patients with advanced disease had exposure to platinum chemotherapy. Patient 4 had the longest treatment period. Initially, she presented as locally advanced and later had recurrence/metastatic disease for which she received systemic therapy with multiple lines. She passed from fatal pneumonitis secondary to immunotherapy.

## Discussion

Patients with DDR-related pancreatic cancer had significantly improved survival in our cohort. This contrasts sharply with historical landmark studies of pancreatic cancer where survival ranges between of 6–11 months [13]. Response duration was also significantly longer compared to what has been reported, likely secondary to increased sensitivity to DNA-damaging drugs. The increased survival is comparable to previous research on DDR-related pancreatic cancer cohorts [2, 12].

Patient 11 was the only individual in this population to pass away before the general population median survival time for a similarly staged tumor. Unfortunately, at the time of her pancreatic cancer diagnosis, she had other significant co-morbidities, including end-stage renal disease requiring dialysis. At the time of her passing, there was no evidence of cancer recurrence on MRI or CT.

Estimates vary, but around 5–15% of all patients with pancreatic cancer have a detectable pathogenic DDR-related gene variant, and around 5% have a *BRCA1/2* variant specifically [14–16]. The National Comprehensive Cancer Network (NCCN) recommends *BRCA1/2* analysis for all diagnosed with pancreatic adenocarcinoma [17]. The significant, potential impact for the patient and their family has led to this approval.

Even with potential, personal benefit, cost can still be a prohibitive factor. Patient 2 had not been able to complete germline testing initially due to high personal cost despite young diagnosis and family history of pancreatic, prostate, and uterine cancer in first degree relatives. Results of circulating tumor DNA (ctDNA) testing and an additional testing platform that reported somatic/ germline status confirmed his germline *BRCA1* variant.

It is less well studied whether other DDR-related gene variants would respond to platinum based chemotherapies and/or PARP-inhibitors in same way as *BRCA1/2*. *BRCA1/2* and *PALB2* are known to be associated with an increased risk for pancreatic cancer [18–21]. Evidence supports that risk for pancreatic cancer may be elevated as well in those with a pathogenic *ATM* variant [22], and *BRCA1* is a downstream target of the *ATM* gene [23]. *FANCC* is less well characterized and associated with lower penetrance for hereditary cancer risk [17]; limited research suggests an association with pancreatic cancer [24, 25]. The *FANCC* gene is a DDR-related gene in the Fanconi anemia pathway [26]. Decisions regarding chemotherapy should be weighed and discussed on an individual basis preferably in a molecular tumor board setting. Further research should include these other DDR-related cohorts to explore if they derive similar benefit. It is also important to note that most experts would suggest that cisplatin may be superior as compared to other platinum drugs. Furthermore, irinotecan, which is part of FOLFIRINOX combination chemotherapy, is a DNA-damaging drug (topoisomerase inhibitor). Therefore, the benefit derived in patients who are exposed to FOLFIRINOX is likely from both the irinotecan and the platinum part of the combination chemotherapy.

The relatively small sample size, large number of resectable tumors, and the retrospective, single-institutional nature of this study with selection bias are all limitations.

## Conclusions

Our study corroborates previous studies and expands the literature with inclusion of non-BRCA1/2 genes. This case series does suggest that patients with pancreatic cancer due to DDR-related genes may have better overall outcomes than the general population with pancreatic cancer. Their response to platinum based or other DNA-damaging chemotherapies may be the driving factor. Similar results are being reported from pooled large cohorts from other major academic centers. With universal germline testing now endorsed for pancreatic cancer, data regarding DDR-related pancreatic cancer will significantly increase. For the time being, with platinum-based therapies already approved for these patients, if there is a choice, it would be reasonable to choose a DNA-damaging based therapy and/or participation in some of the PARP-inhibitor trials.

## Data Availability

The dataset generated/ analyzed during the current study are not publicly available as individual privacy could be compromised but are available from the corresponding author on reasonable request.

## Abbreviations

MMR: Mismatch repair
DDR: DNA damage repair
MSI: Microsatellite instability
PARP: Poly ADP ribose polymerase
VUS: Variant of uncertain significance
NCCN: National Comprehensive Cancer Network
ctDNA: Circulating tumor DNA
